# Supplementary material for: Long-term stability and the physical and chemical factors predictive for antimicrobial activity in Australian honey
Source: PLoS One. 2024 May 22;19(5):e0303095. doi: 10.1371/journal.pone.0303095 (PMC11111008; doi:10.1371/journal.pone.0303095)
Supplement: Attachment — Submitted filename: Response to Reviewers.docx [file pone.0303095.s004.docx]

We thank both reviewers for their helpful comments and suggestions which we have taken on-board to improve the manuscript. We address each of these below.

**Reviewer #1**

Manuscript PONE-D-24-05003 describes mainly the antimicrobial properties of honey samples after they has been stored for 15-17 years. I must say that the experimental section is of very high quality, both in conceptualisation as well as execution. Interpretation of the results is also very thorough, even though some points need attention, as mentioned below. However, the major concern in this manuscript is the scope of the whole work, as well as the importance of the conclusions. At first, the selection of the samples is surprising. Why would anyone choose 15–17-year-old honey samples to conduct so many experiments? What would they want to show? And how this can help industry meet the growing demand for high-quality, medicinally active honey? Does industry market honey that old? In addition, why would anyone be interested in investigating how the antimicrobial activity of honey changes after 15-17 years of storage? Would anyone ever store honey for so long? Also, did you evaluate organoleptic properties of those samples to see how they have changed through time? I understand that scientific curiosity could easily drive someone to do such a research, yet this is far beyond the scope that the authors state or the conclusions they wanted to achieve. It is not mentioned in the Abstract that samples were 15-17-year-old! You conducted an excellent series of experiments and made conclusions with very old honey samples. Why? From where I see it, the authors must either reconsider the scope of this manuscript or provide strong arguments for their choice to employ so old samples in their work.

Thank you for your detailed and thoughtful assessment of our manuscript, and for acknowledging the quality of the experimental design and execution. We recognise your concerns regarding the choice of employing 15–17-year-old samples in our study.

These samples were collected as part of an unusually large and comprehensive survey of Australian honeys conducted over several years. The samples are extremely well-characterised, with detailed information regarding their collection date, floral source assessment, beekeeper storage and processing, and lab storage conditions. The diverse array of samples and level of detail in their characterisation provides an invaluable dataset for understanding both how honey properties correlate with antimicrobial activity, and how antimicrobial properties change over time.

In our lab we have utilised these samples over many years for various projects and have discovered that potent antimicrobial properties remain remarkably stable. While it is true that the industry does not currently market honey as old as 15 years, it is well known that, if stored correctly, honey has a basically infinite shelf life. With the growing burden of expired medicines contributing to environmental contamination and landfill waste, we believe medicinal honey represents an underutilised sustainable alternative.

Building upon your suggestion to reconsider the scope of this manuscript, we have reworked the abstract and introduction to emphasise the exploration of the longevity properties of honey and its potential implications for sustainability as discussed above.

Changes have been made to:

Abstract (Lines 15-21, 24)

Introduction (Lines 41-55, 63, 84-86)

Discussion (Lines 538-545)

Conclusions (Lines 710-716)

Additional major comments

Title. The title does not reflect the content of the manuscript. Is this work about long-term stability and how this is developed in the manuscript? Also, I agree that MIC is predictive for antimicrobial activity, but I believe it cannot be considered a chemical factor.

We believe that the changes to the manuscript as outlined above make the title more appropriate. We consider MIC to be a measure of antimicrobial activity rather than a predictor.

Lines 311-312. The authors here state a major problem in the interpretation of their results. Based on the publication of Irish et al. (2011), honey origin was verified by the apiarists that collect them. This raises a concern regarding the correct assignment of the floral source of the samples. In any case, you should add in Materials and method show the botanical origin of samples was verified.

It is not unexpected that even in primarily “monofloral” honeys there may be traces of other nectar sources. However, the details of floral source assessment have been added to the methods as suggested:

“The floral source of the honey was determined by beekeepers through a process that considered the floral resources available for nectar collection, the geographical location of the apiary, and organoleptic characteristics of the honey.” (Lines 100-102)

Lines 395-412. This section needs more analysis and careful wording. It is evident that elemental analysis of honeys can differentiate samples for their geographical origin as metallic components are correlated with the soil where the plants grow. This is in agreement with the finding that Jarrah honey contains large quantities of several elements. Additionally, it is well known that honeydew honeys contain large quantities of ash compared to flower honeys. I am sorry, but I am not familiar with the Australian flora. Are there any honeydew honeys in this research? This information would be interesting for the reader and could help you better explain your results. The comment that maybe these large quantities of elements could have been introduced during processing and packaging is arbitrary and must either be removed or supported by literature. I agree that this can happen, however few of the elements found in honey can result through this route.

There were no honeydew honeys used in the current study. We have removed the statement re elemental contamination during processing and packaging as we agree it is unsubstantiated (Lines 438-439).

Lines 560-569.These lines seem problematic. Typical water activity of honey is 0.5 to 0.65, while most microorganisms need more than 0.7 to grow. In your work, you measured water activity was as high as 0.69. It is not honey with low water activity that do not favour microbial growth, it is rather all honeys that do so! In addition, the shelf stability of honey is not correlated with microbial inhibition! It is HMF and diastase activity that determine shelf life of honey. Microbes almost never develop in honey, with the exception of osmophilic yeasts under favorable conditions. Moreover, can you determine what you mean with “low water activity” in line 565? In my opinion, shelf stability of honey cannot be correlated with water activity.

We agree that all honeys have low activity and do not favour microbial growth. We have adjusted this section as follows:

“However, as honey is diluted to at least 32% (w/w) in the MIC assay it is unlikely that water activity directly contributes to its antimicrobial properties. Instead, it likely serves as an indicator for other factors that may alter antimicrobial activity. Water activity decreases with the increased bee processing, which could result in higher levels of bee enzymes and antimicrobial peptides. Differences in nectar sources can also affect water activity, and these may introduce other antimicrobial features.” (Lines 595-605).

Minor comments

Line 224. You wrote 2002 instead of 2022.

This has been corrected.

Lines 389-390 and elsewhere. HMF is measured in mg/Kg of honey and ppm is hardly ever used. Even though it is not wrong, someone would be surprised to see it (as I did).

The units ppm have been changed to mg/ kg for MGO, DHA, and HMF in Table 2, Table S1 and the results section of the text.

Lines 646-653. Include the conditions of storage of the referenced works that you include here.

This has been added.

Line 662. How can any compound in honey react with oxygen apart from those on the surface?

We have added : “though this would likely only apply to compounds on the surface.” (Line 702)

Line 667. Predictor, not predicator.

This has been corrected.

**Reviewer #2**

Please see my corrections and comments in the corrected manuscript.

We have incorporated the corrections suggested in the manuscript.

Were the V8-Juice Agar plates also incubated at 35 °C? (The results section made it evident that the answer to this question is affirmative.) This temperature is not suitable for determination of mould counts, except in specific cases. Instead, a temperature of 25 °C should have been used. Incubation of the yeast and mould selective agar plates at lower temperatures (25 °C) may have resulted in more mould growth.

Thank you for pointing this out, we have added a sentence acknowledging this:

Lines 317-318: “… however, incubation of plates at a lower temperature (25 °C) may have resulted in more mould growth.

As each table/figure should be meaningful on its own, without reading the text, please provide the meaning of the abbreviations TA and NPA below the table, as for PE 2022 and MIC (even though this is done in the text of the manuscript).

We have added the meaning of the abbreviations TA and NPA to the footnote below the table.

**Additional Editor Comments**

**I believe the authors failed to spot my comments in the previous review round as they remained unaddressed. Please check under Additional Editor Comments.**

Apologies, we indeed missed the comments in the previous review round. We have now addressed them as below.

The authors are advised to put more effort on vindicating the selection of 15-17 years old honey in the experiments, as required by Reviewer #1. Additionally, the aim of the study in the last paragraph of Introduction should be reinforced.

We have reworked various sections to emphasise the exploration of the longevity properties of honey and its potential implications for sustainability in order to validate the selection of 15-17 year old honey.

Changes have been made to:

Abstract (Lines 15-21, 24)

Introduction (Lines 41-55, 63, 84-86)

Discussion (Lines 538-545)

Conclusions (Lines 710-716)

Please note that Reviewer #2 provided their comments in the attached file.

Thank you, we have addressed these.

Terms in subsections of M&M stand capitalized, while deviation from this rule is seen in Results as well as in Discussion. Please uniform this.

We have removed capitalisation from the subsections in the Materials & Methods section, so all sections are now uniform.

**Other**

We note that some concentrations were erroneously reported as % (w/v) on figures instead of % (w/w) – this has been corrected.
